# Improving poxvirus-mediated antitumor immune responses by deleting viral cGAMP-specific nuclease

**DOI:** 10.1038/s41417-023-00610-5

**Published:** 2023-04-04

**Authors:** Stephanie Riederer, Ana del Canizo, Javier Navas, Marlowe G. Peter, Ellen K. Link, Gerd Sutter, Juan J. Rojas

**Affiliations:** 1 Division of Virology, Department of Veterinary Sciences, LMU Munich, Munich, Germany; 2 Immunology Unit, Department of Pathology and Experimental Therapies, School of Medicine, University of Barcelona—UB, Barcelona, Spain; 3 Immunity, Inflammation, and Cancer Group, Oncobell Program, Institut d’Investigació Biomèdica de Bellvitge—IDIBELL, Hospitalet de Llobregat, Barcelona, Spain; 4 German Center for Infection Research (DZIF), Partner Site Munich, Munich, Germany

**Keywords:** Cancer immunotherapy, Tumour virus infections

## Abstract

cGAMP-specific nucleases (poxins) are a recently described family of proteins dedicated to obstructing cyclic GMP-AMP synthase signaling (cGAS), an important sensor triggered by cytoplasmic viral replication that activates type I interferon (IFN) production. The *B2R* gene of vaccinia viruses (VACV) codes for one of these nucleases. Here, we evaluated the effects of inactivating the VACV B2 nuclease in the context of an oncolytic VACV. VACV are widely used as anti-cancer vectors due to their capacity to activate immune responses directed against tumor antigens. We aimed to elicit robust antitumor immunity by preventing viral inactivation of the cGAS/STING/IRF3 pathway after infection of cancer cells. Activation of such a pathway is associated with a dominant T helper 1 (Th1) cell differentiation of the response, which benefits antitumor outcomes. Deletion of the *B2R* gene resulted in enhanced IRF3 phosphorylation and type I IFN expression after infection of tumor cells, while effective VACV replication remained unimpaired, both in vitro and in vivo. In syngeneic mouse tumor models, the absence of the VACV cGAMP-specific nuclease translated into improved antitumor activity, which was associated with antitumor immunity directed against tumor epitopes.

## Introduction

Oncolytic vaccinia viruses (VACV) are among the most promising oncolytic vectors thanks to their fast and lytic replication cycle in tumors cells and their capacity to stably express one or more transgenes [1]. Importantly, oncolytic VACV can alter the normally immunosuppressive microenvironment of tumors to be more immunogenic, turning “cold” tumors into “hot” ones and acting as an immunotherapy [2]. Due to the release of different danger-associated molecular patterns (DAMPs) and tumor antigens, VACV are able to induce cellular immune responses targeting tumor epitopes [3, 4]. Importantly, the VACV genome can be engineered to express immune-activating proteins [5] or to delete VACV immunomodulatory proteins [6] in order to boost antitumor immunities and improve their therapeutic index.

The presence of foreign DNA in the cytosol is a potent danger signal to the cell, resulting in the induction of innate immune responses [7]. Cytosolic DNA is detected by multiple pattern recognition receptors (PRRs) leading to the production of different chemokines and cytokines, including type I interferons (IFN), as a first line of defense against an impending virus infection [8]. Important DNA sensors that activate type-I IFN production are RNA polymerase III, DNA-dependent activator of IFN-regulatory factors (DAI), DNA protein kinase (DNA-PK), or cyclic GMP-AMP synthase (cGAS) [9–11]. In mammals, cGAS plays a central role in sensing the presence of cytosolic DNA by synthesizing 2’3’-cGAMP (cGAMP hereafter). cGAMP is a cyclic dinucleotide (CDN), molecules that are important second messengers that mediate a wide range of processes in different organisms. This molecule binds to the stimulator of interferon genes (STING), initiating the recruitment of TANK binding kinase 1 (TBK1) and IRF3 activation [7], triggering transcription of genes encoding inflammatory proteins [12].

Although the important role of cGAMP was known, no cytosolic nucleases able to selectively target cGAMP were identified until Eaglesham et al. described the function of the B2 protein in VACV in 2019 [13]. Since then, increasing evidence for the central role played by these cGAMP-specific nucleases in pathogen evasion has been published. In addition to poxviruses, different classes of pathogens have been described to encode proteins with similar functions, including baculovirus [13], cypovirus [14], ribovirus [15], and parasitoid wasps [15].

VACV, as a member of the *Poxviridae* family, is a large double-stranded DNA virus that replicates within specific cytoplasmic compartments, called viral factories [16]. To avoid recognition by the host immune system, poxviruses encode a variety of immunomodulatory proteins to prevent the sensing of cytoplasmic viral DNA [17, 18]. For example, C10 (also called C16 in the Western Reserve strain) blocks dsDNA sensing by binding the Ku subunits of DNA-PK, inhibiting IFN responsive factor 3 (IRF3) activation and IFN production [19]. As mentioned above, another immunomodulatory strategy of VACV is mediated by the recently identified family of poxvirus proteins that inhibit the DNA sensor mediated by cGAS-STING signaling. These nucleases neutralize cGAMP, the cyclic dinucleotide second messenger synthesized when cGAS binds to dsDNA, and were named poxins (poxvirus immune nuclease), which is encoded by the *B2R* gene in VACV.

Since the cGAS pathway plays a central role in sensing cytoplasmic vaccinia DNA and cGAMP-specific nuclease plays an important role in inhibiting this sensing [13, 20], we hypothesized that inactivation of this nuclease in an oncolytic VACV would translate into increased IRF3 phosphorylation and subsequently into increased antitumor cytotoxic T lymphocyte (CTL) responses. Thus, we constructed an oncolytic VACV including a deleted *B2R* gene and evaluated the effects of this deletion both in vitro and in vivo. Our results demonstrated that deletion of *B2R* translates into robust IRF3 pathway activation without affecting viral replication, and in turn this activation translates into improved antitumor activity and increased T-cell responses against tumor antigens in mouse tumor models.

## Materials and methods

### Cell lines and viruses

All cell lines used in this research (MA104, HeLa, B16, CT26 and THP1 cells) were purchased from the American Type Culture Collection (ATCC) and maintained in recommended culture medium containing 5-10% fetal bovine serum and antibiotics at 37 °C, 5% CO_2_. Primary chicken embryo fibroblasts (CEF) were prepared in house from 10-day-old chicken embryos (specific pathogen-free (SPF) eggs, VALO BioMedia, Cuxhaven, Germany). Cell lines were regularly tested for mycoplasma contamination.

The highly attenuated VACV vaccine strain MVA was previously described [21, 22]. All replication-competent recombinant VACV used or constructed in this work are based on the VACV strain Western Reserve (WR). Construction of WR/TK- (VACV strain WR with a truncated viral thymidine kinase (TK) gene and expressing mCherry under the VACV-specific late promoter P11) was previously described [23]. The *B2R* gene was inactivated in WR/TK- by guided homologous recombination replacing the original gene sequence with a synthetic construct containing 350 bp upstream and 350 bp downstream of the *B2R* genomic site. Furthermore, the *B2R* start codon in the synthetic gene sequence was mutated. To induce homologous recombination, a shuttle plasmid containing the synthetic Δ*B2R* construct was transfected into MA104 cells infected with WR/TK− prior to transfection. GFP was used as a reporter to select *B2R*-deleted clones by a positive-negative screening system, and deletion was confirmed by PCR and DNA sequencing. VACV were purified by ultracentrifugation through sucrose cushions as previously described [24, 25] and titered by plaque assays in MA104 cells (for replication-competent VACV) or in CEF cells (for the MVA strain).

### Virus growth and plaque assays

Virus growth was assessed by seeding 2 × 10^5^ cells in 24-well plates and infecting them at a multiplicity of infection (MOI, plaque-forming units [PFU]/cell) of 5 or 0.05. One hour after infection, cultures were washed with PBS and new pre-warmed medium was added. Samples including cells and supernatant were harvested at different time points (0, 4, 12, 24, 48 and 72 h after infection) and frozen at −80 °C. After three freeze-thaw-cycles, viral titer was determined by a plaque assay. Viral yield was evaluated in quadruplicate in two independent experiments.

To determine the size of the plaques, cell cultures were infected at a MOI of 0.05 and 72 h post infection, the diameter of plaques was measured after dyeing with crystal violet. 25 representative plaques per group were measured.

### In vitro cytotoxicity assay

5 × 10^4^ cells were seeded in 96-well plates and infected with 1:5 serial dilutions starting at an MOI of 150 (ranging from 150 to 0.0001) and incubated at 37 °C. Three days after infection, cells were checked for remaining metabolic activity using a non-radioactive cell proliferation assay (CellTiter96® AQueous Non-radioactive cell proliferation assay, Promega, Fitchburg/Wisconsin, USA) following the manufacturer’s instructions. Three different replicates from two independent experiments were evaluated.

### Protein analysis

To detect phosphorylated IRF3 after infection, cells were seeded in 24-well plates and infected at a MOI of 10. Five hours after infection, cells were lysed using RIPA buffer supplemented with 1% protease-phosphatase inhibitor cocktail (Thermo Scientific, Waltham, MA, USA). Equal amounts of protein were separated by SDS-PAGE (10% polyacrylamide) gel electrophoresis and transferred onto a nitrocellulose blotting membrane. After blocking the membrane with TBS/Tween 20 with 5% BSA, a monoclonal anti-p-IRF3 primary antibody (S396 rabbit mAB, Ref 4947, Cell Signaling, Danvers, MA) diluted 1:1000 in TBS/Tween 20 with 1% BSA and a polyclonal anti-rabbit conjugated with HRP (Ref 7074, Cell Signaling, Danvers, MA) diluted 1:5000 in TBS/Tween20 with 1% BSA were used for detection. A rabbit anti-GAPDH-antibody (Ref 2118, Cell Signaling, Danvers, MA) diluted 1:1000 in TBS/Tween 20-1% BSA was used to detect the loading control GAPDH protein. Densitometric analysis was performed using ImageJ software and normalized to GAPDH.

To quantify the amounts of phosphorylated NF-κB, cells were seeded in 24-well plates and infected at a MOI of 10. Five hours after infection, cells were harvested and a NF-κB p65 (pS536) ELISA Kit (Abcam, Cambridge, UK) was used following manufacturer’s instructions. Two technical replicates of four independent samples were evaluated for each cell line.

### RT-pPCR

1 × 10^6^ cells/well were seeded in 24-well plates and infected at a MOI of 5. Three independent replicates were performed for each condition. Six hours after infection, cells were harvested and total RNA was purified using a RNeasy Plus Mini Kit (Qiagen, Hilden, Germany). Remaining genomic DNA was eliminated by digesting the samples with RQ1 RNase-free DNase (Promega, Madison, WI, USA). To synthesize cDNA, an Omniscript RT Kit (Qiagen, Hilden, Germany) was used, and the RT-qPCR was performed using the Luna® Universal qPCR Master Mix (New England Biolabs, Ipswich, MA, USA) under the following conditions: initial denaturation at 95 °C for 1 min, followed by 42 cycles of denaturation at 95 °C for 15 s and annealing/extension at 60 °C for 30 s, followed by melting curve at 65–95 °C, 0.5 °C/cycle. To perform and analyze the qPCRs, the AriaMx real-time PCR system (Agilent Technologies, Santa Clara, CA, USA) and the corresponding Aria 1.7 software were used. Relative expressions of IFN-β were normalized to those of GAPDH (for human cells) or β-actin (for mouse cells) using the 2^-ΔΔCq^ method. The following primers, described previously [26, 27], were used: hIFN-β-F: 5′-GCTTGGATTCCTACAAAGAAGCA-3′, hIFN-β-R: 5′-ATAGATGGTCAATGCGGCGTC-3′, hGAPDH-F: 5′-ATTTGGCTACAGCAACAGG-3′, hGAPDH-R: 5′-TTGAGCACAGGGTACTTTATT-3′, mIFN-β-F: 5′-CAGCTCCAAGAAAGGACGAAC-3′, mIFN-β-R: 5′-GGCAGTGTAACTCTTCTGCAT-3′, mβ-actin-F: 5′-AGTGTGACGTTGACATCCGT-3′, mβ-actin-R: 5′-GCAGCTCAGTAACAGTCCGC-3′.

### Mouse models

All animal experiments were approved either by the Government of Upper Bavaria, Munich, Germany (55.2-2532.18-36) or by the Ethics and Animal Experimentation Committee of the University of Barcelona (57/21). 8-week-old female BALB/c (CT26 tumor model) or C57Bl/6 (B16 tumor model) mice were purchased from Charles River Laboratories and housed in an isolated (ISO) cage unit with free access to food and water. For tumor implantation, tumor cells were trypsinized and 5 × 10^5^ cells were subcutaneously implanted in the flank of mice. When tumors reached a volume of 50–100 mm^3^, cages harboring mice were randomized (block randomization) and viruses were administrated intratumorally. The number of animals necessary for each experiment was calculated using a G*Power software Version 3.1.9.2 and the following settings: one tail, effect size d of 1, alpha error probability of 0.05, a power (1-beta error probability) of 0.8, and an allocation ratio N2/N1 of 1. Animals were excluded from the experiments only in case of defective virus administration. Researchers were not blinded for animal studies, although tumor measurements were performed mainly by animal caretakers.

For depletion experiments, anti-mouse CD8 (2.43, Ref BE0061) and rat IgG2b isotype control (LTF-2, Ref BE0090) were purchased from BioXCell (Lebanon, NH, USA). Mice were injected intraperitoneally with 500 µg of antibodies at days 1 and 2 after tumor implantation, followed by 250 µg injection every 5 days till the end of the experiment.

### Study of viral replication and IFN-β quantification in vivo

Tumors were established as described above. Mice were randomized (*n* = 4-6) and intratumorally injected with a single dose of 1 × 10^7^ PFU. At day 4 after virus injection, mice were sacrificed and tumors were harvested, washed with PBS, and fluorescence signals from tumors were acquired using a Geldoc imaging system (Bio-Rad, Hercules, CA) and quantified using ImageJ.

To determine viral titer and IFN-β concentrations within tumors, mice were treated as described above and tumors were harvested, weighted, and homogenized using metal beads and a tissue homogenizer (Qiagen, Hilden, Germany). Virus titers were determined by plaque assays on MA104 cells and IFN-β was quantified using a mouse IFN-β ELISA kit (R&D Systems, Minneapolis, MN, USA). Six mice per group were used to determine IFN-β concentration.

### In vivo antitumor activity

Tumors were established as described above and mice were injected twice (days 0 and 4) with an intratumoral dose of 1×10^7^ PFU of different viruses. Tumor size was monitored by caliper measurements and defined by V(mm^3^) = π/6 × W^2^ × L, where W and L are the width and the length of the tumor. 14 mice per group were used to determine antitumor activity.

For Kaplan-Meier survival curves, the endpoint was established at ≥750 mm^3^. Animals whose tumor size never achieved the threshold were included as right-censored information.

### IFN-γ ELISpot

Tumors were established as described above and splenocytes were prepared at day 9 after virus administration from mice treated twice with an intratumoral dose of different viruses. 2 × 10^5^ cells were cultured for 48 h in anti-IFN- γ (MABTECH, Stockholm, Sweden) pre-coated 96-well plates together with 2 µg/ml of peptides. The synthetic peptides used for restimulation were: B8R (TSYKFESV), gp100 (KVPRNQDWL) and B16-M30mut (PSKPSFQEFVDWENVSPELNSTD). An automated ELISpot reader (A.EL.VIS Eli.Scan, Hannover, Germany) was used for counting and analyzing. Eight mice per group were used for ELISPOT assays.

### Flow cytometry

Tumors were established as described above and harvested at day 9 after virus administration from mice treated twice with an intratumoral injection of indicated viruses or PBS. Tumors were mechanically disaggregated and digested with collagenase IV and DNase type I (Merck, Darmstadt, Germany). After filtration through 70- and 40-µm nylon cell strainers, mouse Fc receptors were blocked with FcεRIII/II-specific antibody (clone 2.4G2, Ref 553142, BD Biosciences, San Jose, CA, USA). Tumor-disaggregated cells were stained using APC anti-mouse CD8, FITC anti-mouse CD4 (Refs 22150086 and 22150043, Immunotools, Friesoythe, Germany), and LIVE/DEAD Fixable Near IR (780) Viability Kit (Invitrogen, Waltham, MA, USA), and cell surface immunostaining analysis was performed using a FACSCanto II Flow Cytometer (BD Biosciences). Four mice per group were used to determine the percentage of tumor infiltrating lymphocytes.

### Statistical analysis

A standard Student’s t test (two-tailed) or a one-way ANOVA and Tukey’s multiple comparison test were used throughout this work to determine whether there are statistically significant differences between the means of two or three or more groups, respectively. In studying antitumor efficacy, a two-way ANOVA and Bonferroni posttest were chosen to analyze tumor growth curves and a log-rank test was used for survival curves. In all cases, significance was achieved when *p* < 0.05.

## Results

### Generation of an oncolytic vaccinia virus with a deleted viral cGAMP nuclease gene

**Fig. 1:**
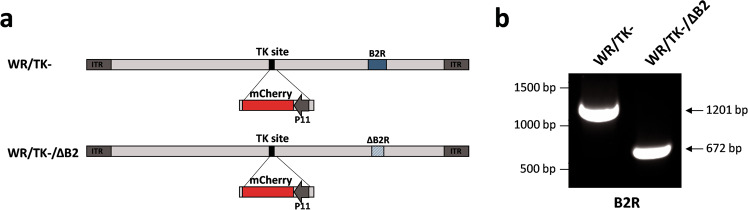
Generation of an oncolytic VACV with a deletion in the *B2R* gene. **a** Schematic diagram of WR/TK- and WR/TK- incorporating a deletion in the *B2R* gene (WR/TK-/ΔB2). To monitor viral replication in tumors, an expression cassette encoding the mCherry fluorescent protein under the control of the viral P11 promoter was inserted into the thymidine kinase (*J2R*) site in the virus genome. **b** PCR analysis to confirm the deletion in the B2R gene. Expected PCR products are: *B2R* = 1201 bp, ∆*B2R* = 672 bp.

We deleted the cGAMP nuclease encoded in VACV by the *B2R* gene in the candidate oncolytic VACV WR/TK− [25] (Western Reserve strain of VACV with a deleted thymidine kinase gene and expressing mCherry to serve as a fluorescence marker of viral gene expression) (Fig. 1a). Complete deletion of *B2R* was confirmed by PCR analysis using oligonucleotides primers flanking the deletion site (Fig. 1b) and by DNA sequencing.

### Replication of the recombinant vaccinia virus in cancer cells was not hindered by loss of cGAMP nuclease

**Fig. 2:**
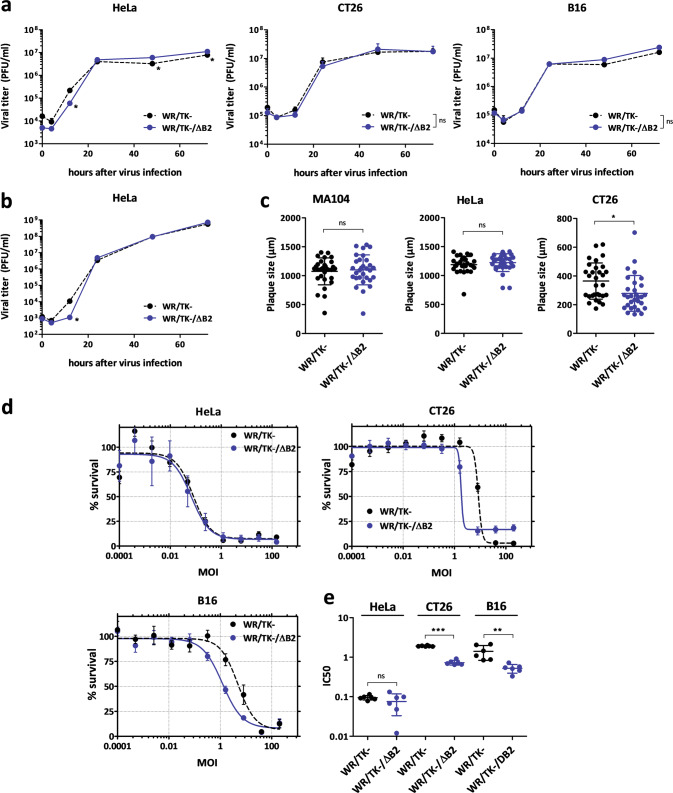
cGAMP nuclease impairment does not have a major impact on viral replication and cancer cell killing capacity. **a**, **b** Robust replication of the B2R-deleted virus. Human and mouse tumor cells were infected with a multiplicity of infection (MOI) of 5 (**a**) or 0.05 (**b**) and at indicated time points, samples were collected and viral titers were determined by plaque assay. Viral yield was evaluated in quadruplicate in two independent experiments. **c** Plaque size. The cell monolayers were infected at a MOI of 0.05 and, 72 h post infection, cells were dyed with crystal violet before measuring the diameter of the plaques. The diameter size (µm) of 25 representative plaques per group and mean ± SD are represented on the dot plot. (**d**, **e**) Comparative cytotoxicity in human and mouse tumor cell lines. Tumor cells were infected with the indicated viruses at doses ranging from 150 to 0.0001 PFU/cell. After 72 h, the % of cells killed (**d**) and IC50 values (**e**) were evaluated (3 different replicates from two independent experiments +SD). *p < 0.05 vs. WR/TK–; **p < 0.01 vs. WR/TK−; **p < 0.001 vs. WR/TK−; ns not significant.

To ascertain whether deletion of *B2R* has an impact on VACV replication and propagation in tumor cells, first we assessed progeny production of WR/TK-/ΔB2 in tumor cells. We analyzed one-step-growth (Fig. 2a) and multiple-step-growth curves (Fig. 2b) compared to those of the control virus WR/TK- after infecting a panel of tumor cells at multiplicities of infection (MOI) of 5 and 0.05, respectively. At indicated time points, cultures were harvested to determine viral titers by plaque assays. In all cancer cell lines tested (HeLa, CT26, and B16), *B2R* deletion had no major impact on viral replication, and only a slight reduction was detected in HeLa cells at 12 h after infection. However, replication levels quickly recovered, and even higher levels of progeny production were detected in this cell line at later time points (Fig. 2a).

Next, we evaluated the size of the virus plaques formed in cell monolayers. In both MA104 and HeLa cells, the plaques formed after infection with WR/TK-/ΔB2 were not significantly different in size compared to those after infection with WR/TK− (Fig. 2c). However, plaque sizes formed in CT26 cells after infection with the *B2R*-deleted virus were slightly smaller. Viral plaque size was not evaluated in B16 cells since they do not support the formation of distinct plaque lesions upon VACV infection.

Although no advantage of B2 inactivation was expected in vitro, *B2R*-deleted oncolytic VACV proved more efficient in killing different cancer cells. We assessed the tumor cell destruction capacity using a metabolic assay after infecting both human (HeLa) and mouse cancer cell lines (CT26 and B16) with MOIs ranging from 0.0001 to 200. 72 h after infection, the remaining metabolic activity of the cells was evaluated (Fig. 2d). We observed a modest improvement of WR/TK-/ΔB2 in CT26 and B16 cells compared to its parental counterpart when the half maximal inhibitory concentrations (IC50) were calculated (Fig. 2e).

### Infection of tumor cells with B2R-deleted oncolytic VACV leads to IRF3 pathway activation

**Fig. 3:**
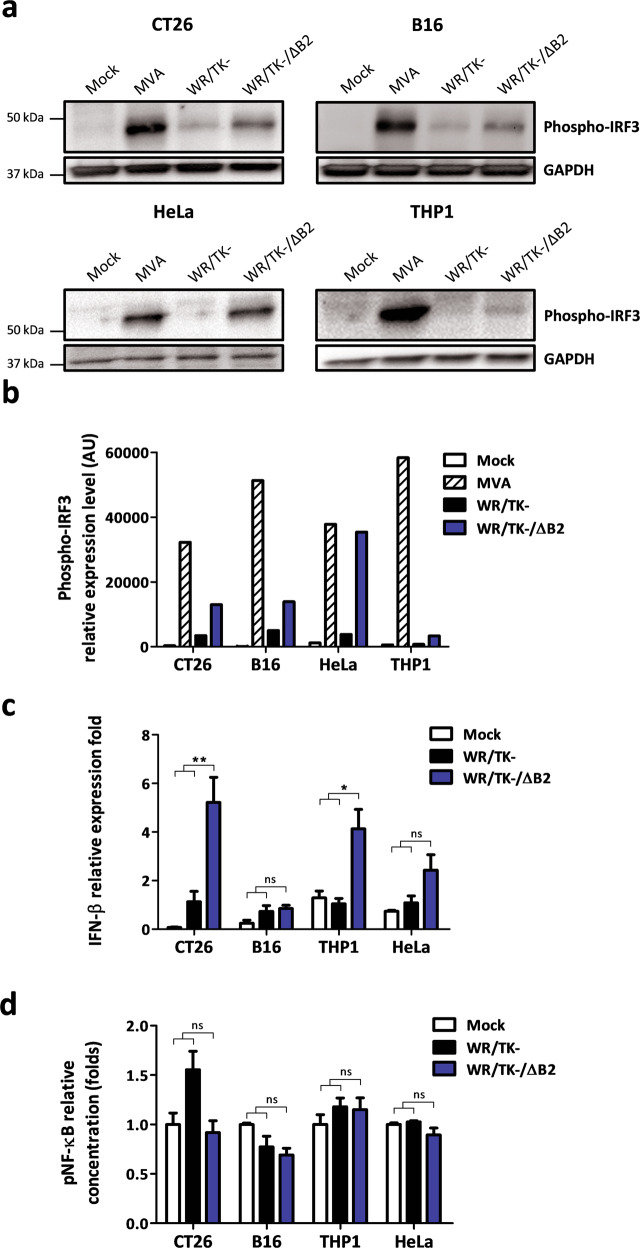
Deletion of *B2R* in oncolytic VACV leads to IRF3 pathway activation. **a**, **b** Phosphorylation of IRF3 in human and mouse cancer cells by candidate oncolytic VACV with a deletion of the *B2R* gene. The indicated cell lines were infected at a MOI of 10 and 5 h after infection, cells were lysed and Western blot analyses were performed using a monoclonal antibody against phospho-IRF3. Mouse phospho-IRF3 and human phosphor-IRF3 have a molecular weight of 45 and 55 kDa, respectively. The replication-deficient VACV MVA served as a positive control and GAPDH detection as a loading control. Two independent experiments were performed and representative Western blot (**a**) and associated densitometric analysis (**b**) are shown. **c** Relative expression of IFN-β measured by RT-qPCR. The indicated mouse and human cancer cells were infected at a MOI of 4 with Mock, WR/TK- or WR/TK-/ΔB2. Total RNA was obtained 6 h after infection. The qPCR data were normalized to the GAPDH gene in HeLa and THP1 cells or β-actin in CT26 and B16 cells by the 2^−ΔΔCq^ method. **d** Activation of NF-κB pathway after infection with WR/TK- and WR/TK-/ΔB2. An ELISA assay was utilized to determine concentrations of pNF-κB in extracts of CT26, B16, THP-1, and HeLa infected at a MOI of 10. Analyses were performed 5 h after infection. Data were obtained in quadruplicate and are plotted as fold change versus Mock + SD. ns not significant; *p < 0.05; **p < 0.01.

We used Western blots to evaluate the levels of phosphorylated IRF3 (P-IRF3) after infection of cancer cells to assess whether the *B2R* deletion affects IRF3 pathway activation in the context of oncolytic VACV infection. Infections with the highly attenuated Modified Vaccinia virus Ankara (MVA) served as a positive control for the activation of the IRF3 pathway; MVA is a natural VACV mutant with many inactivated viral genes, including *B2R*, and is known to efficiently activate IRF3 [21, 28]. Increased levels of P-IRF3 were detected after infection of HeLa, CT26, and B16 cancer cells with WR/TK-/ΔB2 compared to WR/TK- infection (Fig. 3a, b). Upon infection of the human monocyte cell line THP-1, slightly increased levels of P-IRF3 were also detected after infection with the *B2R*-deleted oncolytic VACV.

To confirm activation of the IRF3 pathway, we quantified levels of interferon-β (IFN-β) mRNA upon infection with WR/TK− or WR/TK−/ΔB2 using RT-qPCR. The *B2R*-deleted VACV induced higher levels of IFN-β mRNA in all cell lines tested, which was statistically significant compared to WR/TK- infection in CT26 and THP-1 cells (Fig. 3c).

As another major signaling module used by STING is NF-κB-mediated transcriptional activation [29], we evaluated levels of phosphorylated NF-κB after infection of cancer cells with WR/TK− or WR/TK−/ΔB2. No significant differences in the activation of this pathway were detected using an ELISA assay (Fig. 3d).

### Deletion of viral cGAMP nuclease does not hinder oncolytic VACV replication in tumor models

**Fig. 4:**
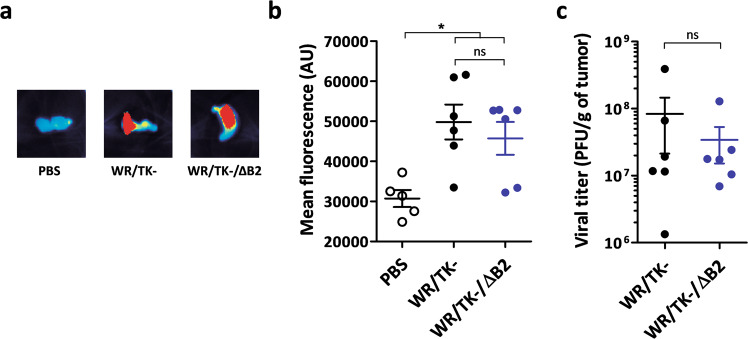
Replication of the cGAMP nuclease mutant VACV is unimpaired in tumor models in vivo. 5 × 10^5^ CT26 cells were subcutaneously implanted on the flank of 8-week-old BALB/C mice (*n* = 5–6). At day 0, a dose of 1 × 10^7^ PFU of the indicated virus was intratumorally injected and 4 days later, mice were sacrificed and the tumors were harvested. **a** Images of representative tumors showing mCherry fluorescence intensity. **b** mCherry fluorescence from the tumors of each group was quantified using a MacroImaging system. Fluorescence intensity (arbitrary units) of individual tumors ±SD is shown. **c** Viral titers determined by plaque assays after tumor homogenization. Titers obtained from each independent tumor and means +SD are plotted on the graph. ns not significant; *p < 0.05.

Although efficient replication of WR/TK−/ΔB2 was demonstrated in vitro, we wanted to assess whether this effective replication is maintained in the complex structure of a tumor. We injected mice bearing CT26 tumors (colon adenocarcinoma) intratumorally with WR/TK− or WR/TK−/ΔB2 and, 4 days after virus injection, tumors were harvested and fluorescence emitting from virus-expressed mCherry within the tumor tissue was quantified. No significant differences were found within tumors injected with the different viruses (Fig. 4a, b). Effective replication of WR/TK− and WR/TK−/ΔB2 was corroborated by titrating virus loads after harvesting and homogenizing the tumors 4 days after virus administration (Fig. 4c).

### Enhanced antitumor activity of oncolytic VACV with a deleted cGAMP-specific nuclease

**Fig. 5:**
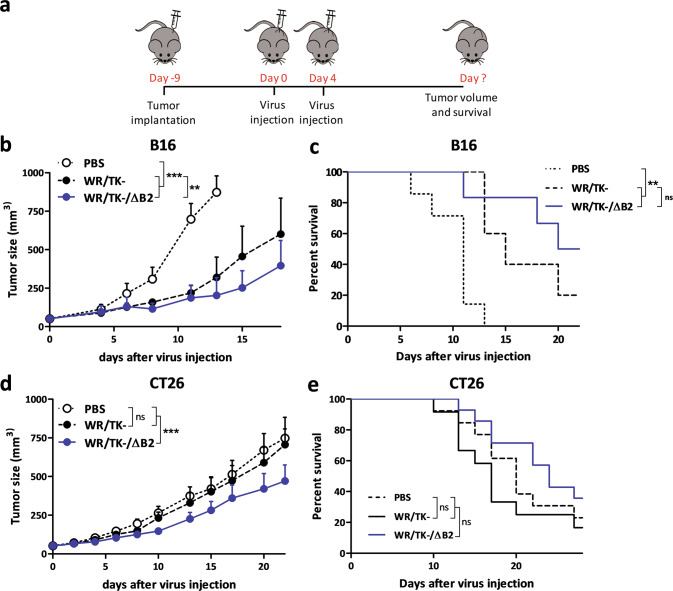
Oncolytic VACV with a deleted cGAMP nuclease shows increased antitumor activity in mouse tumor models. **a** Scheme of the treatment schedule. 5 × 10^5^ B16 (**b**, **c**) or CT26 (**d**, **e**) tumor cells were subcutaneously implanted at day −9 in the flank of 8-week-old C57Bl/6 (B16 tumors) or BALB/C (CT26 tumors) mice, and viruses were intratumorally administrated at days 0 and 4 at a dose of 1 × 10^7^ PFU/injection. PBS injected mice were used as the control group. To monitor tumor growth, the tumors were measured 2–3 times per week until they reached the termination criteria (≥750 mm^3^; see Materials and methods). Tumor volume (**b**, **d**) and overall survival (**c**, **e**) are plotted for 9–14 mice per group +SEM. Data shown in (**b**) for the control groups PBS and WR/TK– is the same as published in [23] as both studies were done in the same experiment following the principle of the 3Rs. *p < 0.05; **p < 0.01, ***p < 0.001.

Two syngeneic mouse tumor models, BALB/C or C57/BL6 mice bearing CT26 or B16 tumors, respectively, were used to evaluate the antitumor efficacy of WR/TK−/ΔB2 (Fig. 5a). In both models, intratumor administration of WR/TK−/ΔB2 virus resulted in a significant reduction in tumor growth compared with mock and parental WR/TK− controls (Fig. 5b, d). Survival of mice was not increased significantly when injected with WR/TK−/ΔB2 compared to the control virus WR/TK− (Fig. 5c, e).

**Fig. 6:**
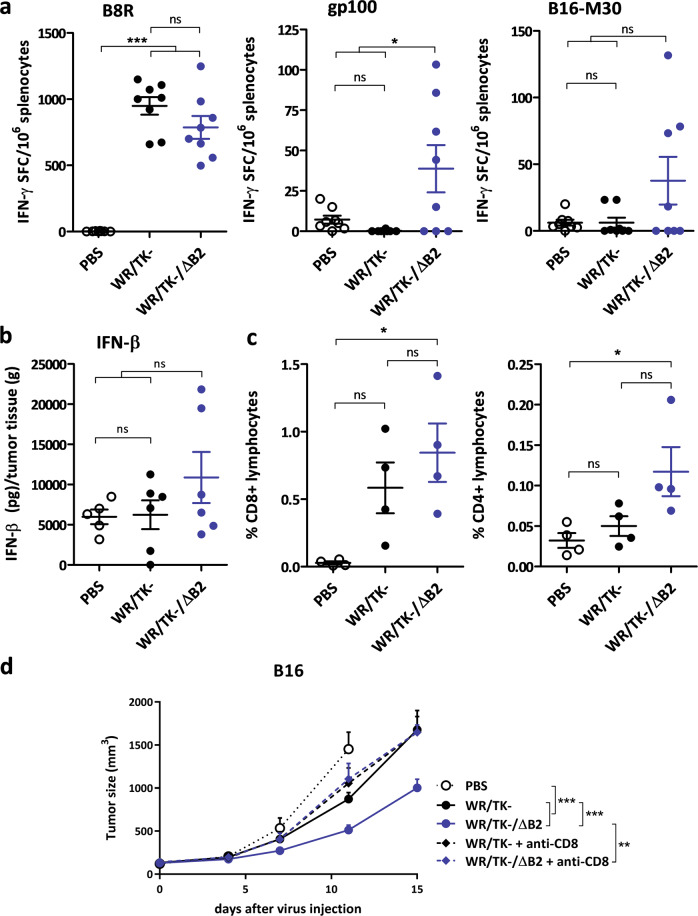
Deletion of the cGAMP nuclease elicits antitumor T-cell responses directed against tumor antigens. **a** Cellular immune response evaluated by IFN-γ ELISpot assay. C57Bl/6 mice harboring B16 tumors were treated as indicated in Fig. 5 and 8 days after virus administration splenocytes were prepared, stimulated in vitro with the indicated peptides, and analyzed for IFN-γ producing cells by ELISPOT. Individual values of IFN-γ spot forming cells (SPC)/10^6^ splenocytes in 8 mice/group and means ± SD are plotted on the graphs. **b** Intratumoral IFN-β concentrations after VACV administration. BALB/C mice harboring CT26 tumors were injected intratumorally with a single dose of 1 × 10^7^ PFU and 4 days later, tumors were harvested and homogenized. IFN-β concentrations were measured using an ELISA assay. **c** Treatment with WR/TK-/ΔB2 increases the number of CD8^+^ and CD4^+^ lymphocytes infiltrating the tumor. C57Bl/6 mice with subcutaneous B16 tumors were treated as before (Fig. 5) and tumors were harvested at day 8 after virus administration and evaluated for lymphocyte populations by flow cytometry. Percentage of CD8^+^ and CD4^+^ live lymphocytes are plotted. **d** CD8^+^ cells are essential for the antitumor efficacy mediated by WR/TK−/ΔB2. C57Bl/6 mice with subcutaneous B16 tumors were injected with CD8-depleting antibodies or isotype control and treated as before (Fig. 5). Tumor volume is plotted for 8–11 mice per group +SEM. ns not significant; *p < 0,05; **p < 0.01; ***p < 0.001.

To ascertain whether deletion of poxviral cGAMP nuclease translates into improved antitumor cellular immunity, we evaluated the tumor epitope-specific T-cell responses established following virus administration in the B16 tumor model using ELISpot assays. Interestingly, T-cell responses directed against the immunodominant VACV-specific B8R peptide epitope [30] were not strengthened when the poxviral cGAMP nuclease is deleted. However, WR/TK-/ΔB2 significantly increased T-cell reactivity responses directed against the tumor-associated antigen gp100 [31] (Fig. 6a). In addition, reactivity against the tumor neoepitope B16-M30 [32] was also increased, although the difference was not significant. When IFN-β within tumors was evaluated by ELISA 4 days after virus administration, concentrations in tumors from mice administrated with WR/TK-/ΔB2 were 2-fold greater than levels in in tumors from mice administrated with the parental WR/TK−, although the difference was not significant (Fig. 6b). Furthermore, when CD8^+^ and CD4^+^ T-cell populations in tumors were quantified by flow cytometry, a significant percentage increase was observed in both populations after treatment with WR/TK-/ΔB2 relative to tumors treated with PBS (Fig. 6c).

To further demonstrate that the enhanced antitumor activity of WR/TK−/ΔB2 is immune-mediated, we evaluated whether depletion of CD8^+^ T cells has an impact on antitumor efficacy. CD8^+^ T cells appeared responsible for such an improvement as their depletion translated into a loss of WR/TK−/ΔB2-mediated antitumor efficacy (Fig. 6d).

## Discussion

Due to the central role of cGAS-cGAMP in sensing cytoplasmic DNA, we hypothesized that infection of cancer cells by an oncolytic VACV including a deleted *B2R* gene could result in more immunogenicity and boost innate immunity within the tumor. Consequently, increased attraction and activation of antitumor cellular immunity would result in viral immunotherapy with better antitumor efficacy. Indeed, our findings support this hypothesis: infection of tumor cells, both in vitro and in vivo, with a VACV containing deletions in the thymidine kinase (TK, *J2R*) and cGAMP nuclease (poxin, *B2R*) genes resulted in activation of the IRF3 pathway, triggering transcription of genes encoding inflammatory proteins, particularly type I interferons. This activation translates into increased numbers of CD8^+^ and CD4^+^ lymphocytes infiltrating the tumor, more robust antitumor cellular responses targeting tumor antigens, and into improved antitumor activity. Importantly, deletion of the cGAMP nuclease does not impair effective VACV replication in either tumor cell lines or tumor mouse models. Previously, it was described that a VACV including a single deletion in the *B2R* gene replicates efficiently in Vero cells [13]. Here, we demonstrate that a VACV combining this deletion with the TK deletion maintains this efficient replication in a panel of tumor cell lines. However, due to the complex structure of tumors and interactions between different kinds of cells composing tumors, in vitro features may not properly reflect the behavior of oncolytic viruses in animal models; but titration of virus load within tumors confirmed effective replication. This was salient since we previously demonstrated the importance of VACV replication to activate effective antitumor immune responses [3].

As already indicated, we deleted the TK gene in our replication-competent VACV in order to achieve selective replication in cancer cells, which is a well-known safety factor since it reduces virus DNA replication and thus virus virulence [33]. Cellular TK, which can functionally complement poxvirus TK, is strongly upregulated in replicating cells, particularly tumor cells, compared to non-dividing normal cells [34]. Thus, TK− viruses can efficiently propagate in many cancer cells and the TK deletion in VACV was the first virus modification introduced into oncolytic VACV (here called WR/TK−) [35].

Besides cGAMP-specific nuclease, VACV encodes a wide variety of immunomodulatory proteins interfering with different innate response pathways that culminate in IRF3 activation. For instance, A46 binds and inhibits TRIF [36], B19 is a soluble type I interferon receptor [37], and K7 binds DDX3 [38]. Previously, we attempted to activate the IRF3 pathway by deleting several of these proteins [23]. We demonstrated that combining a deleted thymidine kinase gene with truncations in immunomodulatory proteins C6 (interacts with NAP1, TANK, and SINTBAD [39]), N2 (blocks nuclear translocation of P-IRF3 [40]), or C10 (prevents dsDNA recognition by DNA-PK [41]) did not translate into IRF3 phosphorylation after cell infection; these three deletions needed to be combined in one virus (named WR/TK-/3Δ) in order detect IRF3 activation and subsequent type I interferon expression. Here, a single deletion in the *B2R* gene was sufficient to achieve activation levels comparable to those induced by the triple-deletion WR/TK-/3Δ virus, further indicating the important inhibitory role played by cGAMP-specific nucleases. In addition, both WR/TK-/3Δ and WR/TK-/ΔB2 viruses, plus a previously described oncolytic VACV expressing TRIF [25], demonstrate a superior capacity to induce antitumor cellular responses and improve antitumor efficacy, supporting our overall strategy of improving viral immunotherapies through (re)activating the IRF3 pathway.

Interestingly, deletion of cGAMP-specific nuclease did not translate into an increase of NF-κB activation. Although stimulation of cGAS-STING promotes canonical and non-canonical NF-κB responses [42], VACV codifies for up to 18 genes interfering with NF-κB signaling. These immunomodulatory VACV proteins include K7 and A52, which bind to IRAK2 and TRAF6 [43], B14, which inhibits IκBα phosphorylation [44], M2, which prevents NF-κB nuclear translocation [45], or K1, which prevents acetylation of the RelA subunit of NF-κB [46]. We hypothesize that the action of these still operating modulators in the context of WR/TK−/ΔB2 infection efficiently inhibits NF-κB activation.

Our findings indicate that deleting the *B2R* gene is a very useful modification to include in a VACV clinical candidate. In addition to improving antitumor efficacy on its own, this deletion could easily be combined with other modifications in order to design candidate viruses with even better capacity to activate efficient antitumor immune responses. For instance, combining expression of cytokines or chemokines (such as GM-CSF [47], IL-12 [48], or CCL5 [49]) could help increase infiltration and activation of T- or NK-cells. Lately, the importance of immunogenic cell death (ICD) for killing cancer cells has emerged, and expression of proteins that activate this kind of cell death (such as MLKL [3]) or co-administration with molecules that enhance ICD (such as vitamin C [50]) could also result in a better antitumor outcome. Similarly, combination with immune checkpoint blockade, either by co-administering [51] or encoding the inhibitor within the VACV genome [52], could also highly benefit the outcome of the therapy. Should one want to accommodate expression of different transgenes listed above to improve different aspects of the antitumor mechanism, then the additional insertion site that the deletion of the *B2R* gene provides could be invaluable. The VACV genome is densely packed with coding regions and few defined loci exist to insert transgenes without compromising viral gene expression and genetic stability of recombinant viruses.

Alternatively, deletion of further VACV genes involved in blocking activation of the cGAS/STING/IRF3 pathway could also maximize the antitumor effect caused by a *B2R* deletion. Notably, the cGAMP nuclease is also deleted in the highly immunogenic VACV strain MVA [13], and we observed that this strain activates the IRF3 pathway better than our candidate virus WR/TK−/ΔB2. In addition to *B2R*, MVA also includes deletions and mutations in several genes involved in blocking IRF3 phosphorylation. Mimicking these deletions in the context of an oncolytic virus could be beneficial to activate IRF3 and subsequently establish antitumor immunity. However, as in MVA, accumulation of these deletions may severely compromise the capacity of VACV to replicate in cancer cells, limiting their lysis and antitumor activity.

Our findings show that deletion of the cGAMP-specific nuclease gene (*B2R*) in oncolytic VACV results in improved antitumor responses mediated by immune responses directed against tumor epitopes. The prominent benefit observed by this single gene deletion bodes well for its combination with assorted genetic modifications to construct different oncolytic VACV clinical candidates with a real potential to produce more reliable and robust responses in a variety of solid tumors.

## Data Availability

The data presented in this study are available on request from the corresponding authors.
